# Evaluation of a new score associated with acute kidney injury in patients treated with cisplatin based EXTREME regimen

**DOI:** 10.1186/s12885-024-12157-1

**Published:** 2024-04-02

**Authors:** François Avry, Charles Roseau, Zoé Leguay, Sixtine Brabant, Alexandre Ganea, Elise Champeaux-Orange, Véronique Priou

**Affiliations:** 1 Centre Régional Hospitalier Universitaire de Tours, 2 Boulevard Tonnellé, Tours, 37000 France; 2 Centre Régional Hospitalier d’Orléans, Orléans, France; 3 Département d’Information Médicale, Centre Hospitalier Régional, Orléans, France; 4 Centre Hospitalier Régional, Service de dialyse et de Néphrologie, Orléans, France; 5 Centre Hospitalier Régional, Service d’Oncologie et de Radiothérapie, Orléans, France

**Keywords:** Cisplatin, Cetuximab, EXTREME, Acute kidney injury, Head and neck cancer, Diuresis, ${U}_{sen}$

## Abstract

**Background:**

This study evaluates the association of diuresis and hydration through a new monitoring indicator called ${U}_{sen}$ and the risk of acute kidney injury in patients treated with cisplatin based-EXTREME regimen.

**Methods:**

We retrospectively reviewed all the cycles of patients with recurrent and/or metastatic head and neck cancer who received cisplatin based-EXTREME regimen from June 2008 to July 2022. Hydration regimen, urine output and concomitant treatments data were collected on the day of cisplatin infusion and the following day of each course received.

**Results:**

Of the 110 courses received by 46 patients, 38 (34.5%) results in AKI. No patient characteristics showed a significant difference between AKI (70%) and non-AKI (30%) group. In univariate analysis, dose reduction of cisplatin (odds ratio = 0.166 [0.04; 0.75], *p* = 0.01)) and ${U}_{sen}$ >8 (odds ratio = 0.316 [0.133; 0.755], *p* = 0.015) and cardiac treatments (odds ratio = 3.24 [1.26; 8.52], *p* = 0.02) were significantly associated with AKI risk. In multivariate analysis, cisplatin dose reduction (odds ratio = 0.129 [0.0241; 0.687], *p* = 0.016) and ${U}_{sen}$ >8 (odds ratio = 0.184 [0.0648; 0.523], *p* = 0.0015) were associated with a risk reduction of cisplatin-related AKI. Concomitant administration of cardiac treatments (odds ratio = 3.18 [1.1; 9.22], *p* = 0.033) showed an increased risk of cisplatin-related AKI.

**Conclusion:**

The combination of diuresis and i.v. hydration through the ${U}_{sen}$ composite score was shown to be associated with cisplatin-induced AKI risk in patients treated with cisplatin based EXTREME regimen. It could be used as a practical indicator to trigger specific clinical management to limit the risk of cisplatin induced AKI.

## Background

Head and Neck Squamous Cell Carcinomas is the sixth most common cancer worldwide with a predicted rising incidence [1]. It is even higher in countries where exposure to certain toxins (mainly alcohol and tobacco) and viral infections (HPV and EBV) is substantial [2, 3]. Although overall survival has increased to 50–68% in recent decades [4, 5], Head and Neck Squamous Cell Carcinoma (HNSCC) remains one of the cancers with the highest relapse rate [6], especially in locally advanced cases with at least 50% of patients developing 2 years treatment locoregional or distant recurrence [7–9]. For patients with locoregional failures, salvage surgery is considered as the best treatment option [10], but the low eligibility rate (about 20–30%) and high risk of second recurrence [11, 12] make the recurrent and/or metastatic (R/M) HNSCC overall prognosis poor (with median survival ranging from 6–12 months) [13]. Thus, these patients are mostly eligible for palliative therapies.

Since the KEYNOTE-048 clinical trial, the combination of pembrolizumab, platinium and 5-Fluorouracil (5FU) is considered as the preferred first line option for all patients surgically or radiotherapeutically ineligible presenting R/M HNSCC [10]. Better tolerability and superior overall survival rate have been shown compared to the EXTREME regimen [14]. This previous standard treatment remains nevertheless a first line option and seems certainly valuable in patients with a PD-L1 combined positive score (CPS) < 1 and/or substantial HNSCC loco-regional recurrence [14, 15].

The EXTREME regimen is composed of cetuximab, a chimeric mouse–human antibody that binds with high affinity to the extracellular of EGFR, either high dose cisplatin or carboplatin and an infusion of 5FU every 21 days [16] (Table 1). The use of carboplatin or cisplatin is left to the discretion and appreciation of the prescriber, considering the higher toxicity of cisplatin [17] but also its superior overall survival benefit in cisplatin subgroup [16].

**Table 1:** Description of EXTREME regimen cycle

Treatments	Dose	Day
Cisplatin	100 mg/m^2^	1
or	or	
Carboplatin	AUC of 5 mg/ml/min	1
Cetuximab	400 mg/m^2^ initial dose then 250 mg/m^2^	1 (first cycle) 1, 8, 15
5FU	1000 mg/m^2^	1, 2, 3, 4

Cisplatin is a well-known antineoplastic agent to have both cumulative and acute nephrotoxicity. Cisplatin is eliminated mainly by glomerular filtration and to a lesser extent by secretion mediate by Organic Cations Transporters 2 (OCT2) in the basolateral membrane side (uptake) and by Multidrug and Toxin Exclusion 1 and 2-K (MATE1/2K) in the apical membrane side (efflux) of renal peritubular proximal cell (RPCs) [18]. Cisplatin RPCs disproportional accumulation is associated with kidney tubular cells damages [19], inducing acute Kidney Injury (AKI) and ionic leakage [20]. If cisplatin-induced AKI generally resolves in few weeks [21], it leads to an increased mortality rate per years [22] and risk of developing or worsening chronic kidney impairment [23].

Associated with cisplatin in the EXTREME regimen, cetuximab is considered as one of the targeted agents presenting the most kindey impairment risk and involving a significant rate of hypomagnesaemia [24, 25] after injection. Initial hypomagnesemia [26, 27], in the same way as high cisplatin dose (> 75 mg/m^2^) [28, 29] are reported to increase the risk of cisplatin-induced AKI. Thus, the combination in the EXTREME regimen is at high-risk of ionic disorders and kidney failure.

Considering the high cisplatin renal uptake [19, 30], limiting nephrotoxicity involves mitigating cisplatin accumulation in the RPCs. Cisplatin Summary of Product Characteristics [31] and clinical recommendations support hyperhydration and ionic supplementation [32–35] to prevent cisplatin-related AKI. For ≥ 100mg/m^2^ cisplatin dose, the optimal hydration regimen is composed of 1L to 1.5L of isotonic saline solution combined with magnesium supplementation 8 to 12 h before the administration of cisplatin followed by at least 2-3L per 24 h to maintain a sufficient diuresis of 3 to 4L the following days. Considering 60 to 80% of the French population is below the daily recommended hydration intake [36, 37] (2L for men and 1.6L for women [38]), it is very likely R/M HNSCCs presenting patients well known for hydration and nutrition disorders (including impaired swallowing, limited mouth opening or diarrhea) are not properly hydrated before the hyperhydration regimen. Moreover substantial alcohol [39]and tobacco [40] consumption and history of chemotherapy treatments put R/M HNSCCs patients at high risk of sub-clinical kidneys, hepatic or cardiac impairments. Indeed, despite high ionic and intravenous hydration, cisplatin kidney failures still highly occur in HNSCCs population [23, 41, 42] and patients are often switched to another treatment line, resulting in a potential loss of healing opportunities.

There, we aim to evaluate via the introduction of a composite variable the association between diuresis, hydrate and AKI risk in patients treated with the cisplatin associated EXTREME regimen.

## Methods

### Patient population and cycles

We retrospectively analyzed the courses of all patients treated with cisplatin-based EXTREME regimen for various head and neck tumors at our hospital center between June 2009 and July 2022. We considered that each as independent, given that the cumulative dose did not appear to influence the risk of AKI [43], that patient management was similar between each course and that the regimen timeframe was short for each patient (negligible effect of age on kidney function).

The cisplatin based EXTREME regimen was administered with concomitant isotonic saline hydration + magnesium, + potassium, ± calcium and ± phosphate supplementation and antiemetic protocol combined aprepitant, corticosteroids (methylprednisolone), ± ondansetron, ± anti-D2 and ± anti-allergic treatment (dexchlorpheniramine). Magnesium supplementation was composed by 1500 mg of i.v. magnesium sulfate at least over the D1.

### Follow-up and study endpoints

Whereas cisplatin induced AKI occur typically 2–10 days after the administration, some happen up to 14 days after the cisplatin treatment. We considered the maximum value of serum creatinine (sCr) within 14 days after the cycle date to evaluate AKI [28]. Baseline creatinine level was defined as the sCr value calculated < 72h before each course. AKI was defined using the National Cancer Institute Common Terminology Criteria for Adverse Events (ver. 4.0). We considered AKI as a ≥ grade 1 acute kidney injury (Creatinine level increase of > 0.3 mg/dL; creatinine level 1.5 -2.0 × above baseline). Patients’ cycles which did and did not meet this definition were placed in the AKI and non-AKI groups, respectively. Patients previously diagnosed with chronic kidney disease (≥ grade 2) were excluded.

The daily hydration and diuresis measured in 8-h increments values are defined according to the following considerations:

- 1^st^ course’s day

It is defined by the interval between the beginning of the patient’s stay and 8am the next day or from 8am on the day of the course to 8am the next day if the patient is already hospitalized.

- Following days

They are defined by 24-h intervals from 8am of each following day. If the patient is no longer hospitalized, the value is defined by the interval from 8am until his or her return home.

We developed a composite variable to assess both the urine volume and its response to i.v. hydration regimen injected to the patient.

Its expression is:

$${U}_{sen}={UV}_{D1+D2}-\left({HV}_{D1+D2}-{UV}_{D1+D2}\right)= {2 \times UV}_{D1+D2}- {HV}_{D1+D2}$$

With:

${U}_{sen}$: Urinal sensibility factor (L)

$${UV}_{D1+D2}$$: D1 + D2 urine output (L)

$${HV}_{D1+D2}$$: D1 + D2 i.v. hydration volume (L)

Initial primary tumor, patient characteristics at each course, chemotherapy modalities were collected and analyzed in search of associate factors of AKI. We included: history of cisplatin treatment before EXTREME regimen, smoking and alcohol status, body mass index during the cycles, cumulative dose, cisplatin and cetuximab dose reduction of each course, age, gender, number of cycles, hepatic impairment (including liver cirrhosis, hepatic dysmorphia, hepatic dysfunction), diabetes mellitus, cardiovascular impairment (including hypertension, heart failure, history of obliterative arteriopathy of the lower limbs and ischemic heart disease), protidemia, kalemia, diuresis and i.v. hydration volume on D1 and D2.

Co-administered treatments were collected on the day of the cycle (D1) and on the following day (D2) as drug potentially associated with nephrotoxicity as NSAIDs, antibiotics (aminoglycosides, glycopeptides or others class of antibiotics) and drugs potentially influencing directly or indirectly cisplatin elimination and/or cisplatin nephrotoxicity.

The primary endpoint is to evaluate the association between ${U}_{sen}$ and the cisplatin induced AKI risk to identify cycles at highest risk of AKI. Secondary endpoints assess clinical (as diuresis), biological data and treatment association (as hydration) concomitant to the cisplatin cycle and AKI risk.

### Statistical analysis

Statistical description and univariate analyses were performed using the online application EasyMedStat and R Software (version R-4.2.0). The methods used were Chi-squared test or Fischer’s exact test (for the relationship between pairs of categorical variables) and the Wilcoxon-Mann–Whitney test or Student t-test (in case of a continuous variable) according to data distribution.

Cut-off value for the classification of urine output (> 7L/48 h) and ${U}_{sen}$ (> 8L/48 h) were determined by a multidisciplinary committee in accordance with current recommendations [31, 32]. A multivariate logistic regression was performed to assess the relation between AKI and the explanatory variables. Variables with a *p*-value < 0.1 in univariate logistic analysis were included in the model. A *p*-value < 0.05 was considered statistically significant.

## Results

**Fig. 1:**
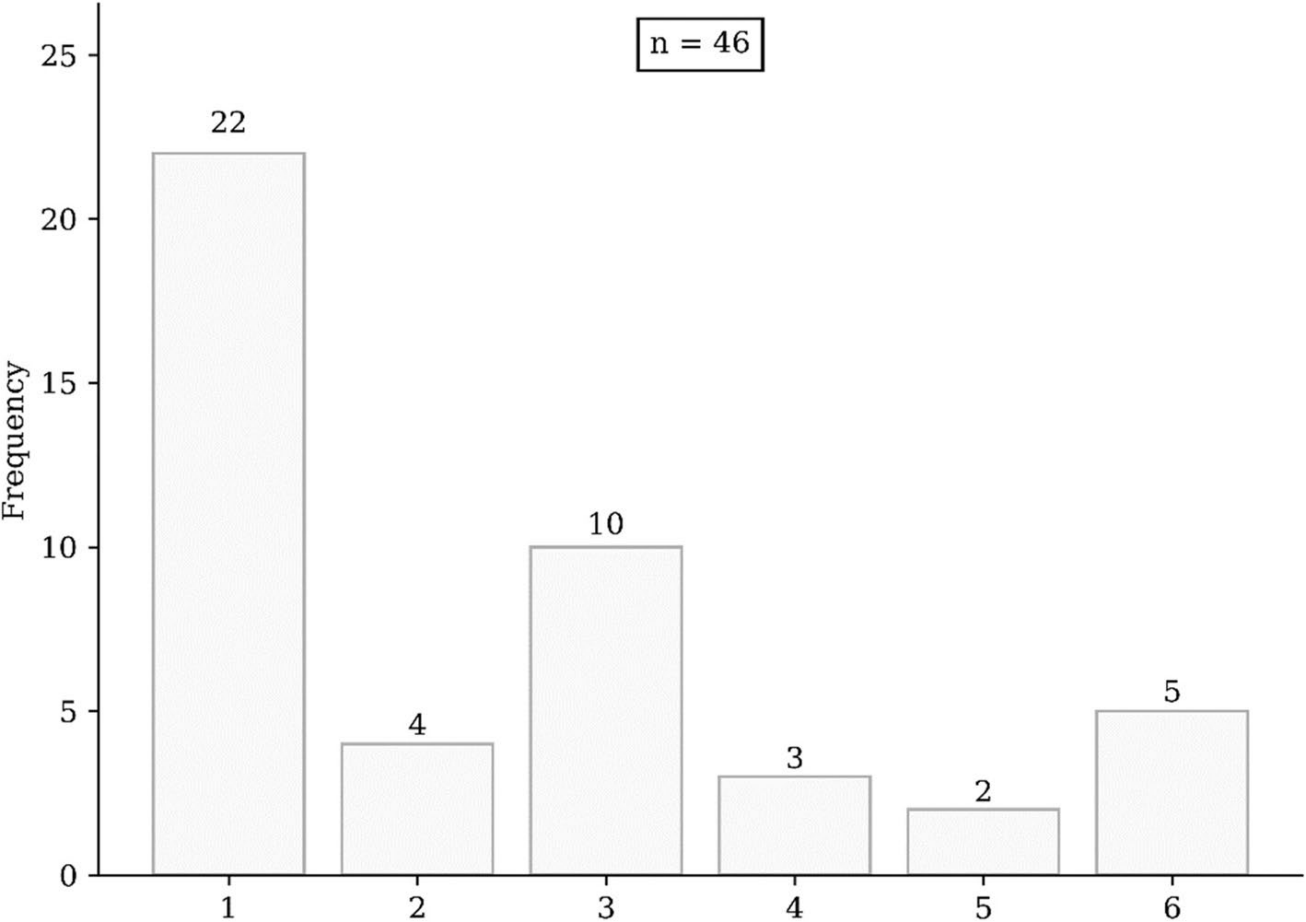
Cycle distribution received per patient

Forty-six patients were included twenty-nine males and seventeen females with a mean age of 60 years (Table 2). 70% of them developed at least one AKI, including 28% after the first cycle. Patients received a median number of cycles of 2 [1-3] (Fig. 1). 85% present alcohol and/or tobacco abuse or abused and 61% present both. There was no significant difference between the two groups regarding patient characteristics.

**Table 2:** Characteristics of patients with and without AKI

	All (*n* = 46)	AKI patients (*n* = 32)	no AKI patients (*n* = 14)	*p*
Age mean (± standard deviation)	60 (± 8.6)	59.5 (8.6)	61.5 (8.7)	0.36
Male, n	29 (63%)	21 (66%)	8 (57%)	0.74
Cisplatin administrated, mean (± standard deviation)	166.2 (22.6)	168.8 (20.8)	160.9 (27.0)	0.3
Number of cycles, median [Q25-75]	2 [1–3]	2.5 [1–3]	1.5 [1–2.75]	0.27
Cisplatin cumulative dose, mean (± standard deviation)	386.5 (276.8)	406.8 (262.8)	340.3 (311.7)	0.46
History of alcohol (> 4U/day) and tobacco (≥ 10 pack-years) abuse, n	28 (61%)	20 (63%)	8 (57%)	0.75
No history of alcohol and tobacco abuse, n	7 (15%)	5 (16%)	2 (14%)	1
Cisplatin anteriority, n	7 (15%)	7 (22%)	0 (0%)	0.083
Diabetes, n	1 (2%)	1 (3%)	0 (0%)	1
Hypertension, n	15 (33%)	12 (38%)	3 (21%)	0.33
Ischemic heart disease, n	4 (9%)	3 (10%)	1 (3.1%)	0.078
Obliterative arteriopathy of the lower limbs, n	4 (9%)	2 (6.2%)	2 (14%)	0.57
Obstructive pulmonary disease, n	2 (4%)	1 (3.1%)	1 (7.1%)	0.52
Liver cirrhosis, n	3 (7%)	3 (9.4%)	0 (0%)	0.54
Male, n	29 (63%)	21 (66%)	8 (57%)	0.74
Meta yes, n	23 (50%)	14 (44%)	9 (64%)	0.34
Radiotherapy, n	40 (87%)	28 (88%)	12 (86%)	1
Initial tumor status, n				
N, n				0.47
N0	6 (13%)	3 (9.4%)	3 (21%)	
N1	8 (17%)	6 (19%)	2 (14%)	
N2	23 (50%)	17 (53%)	6 (43%)	
N3	9 (20%)	6 (19%)	3 (21%)	
N4	0 (0%)	0 (0%)	0 (0%)	
T, n				0.32
T1	1 (2%)	1 (2%)	0 (0%)	
T2	14 (30%)	11 (34%)	3 (21%)	
T3	13 (28%)	9 (28%)	4 (29%)	
T4	18 (39%)	10 (31%)	8 (57%)	
Initial cancer location, n				0.49
oropharynx	20 (44%)	15 (47%)	5 (36%)	
larynx	16 (35%)	10 (31%)	6 (43%)	
oral cavity	7 (15%)	3 (9%)	4 (29%)	
hypopharynx	2 (4%)	2 (6.2%)	0 (0%)	
nasopharynx	2 (4%)	2 (6.2%)	0 (0%)	
nasosinus	3 (7%)	3 (9%)	0 (0%)

**Fig. 2:**
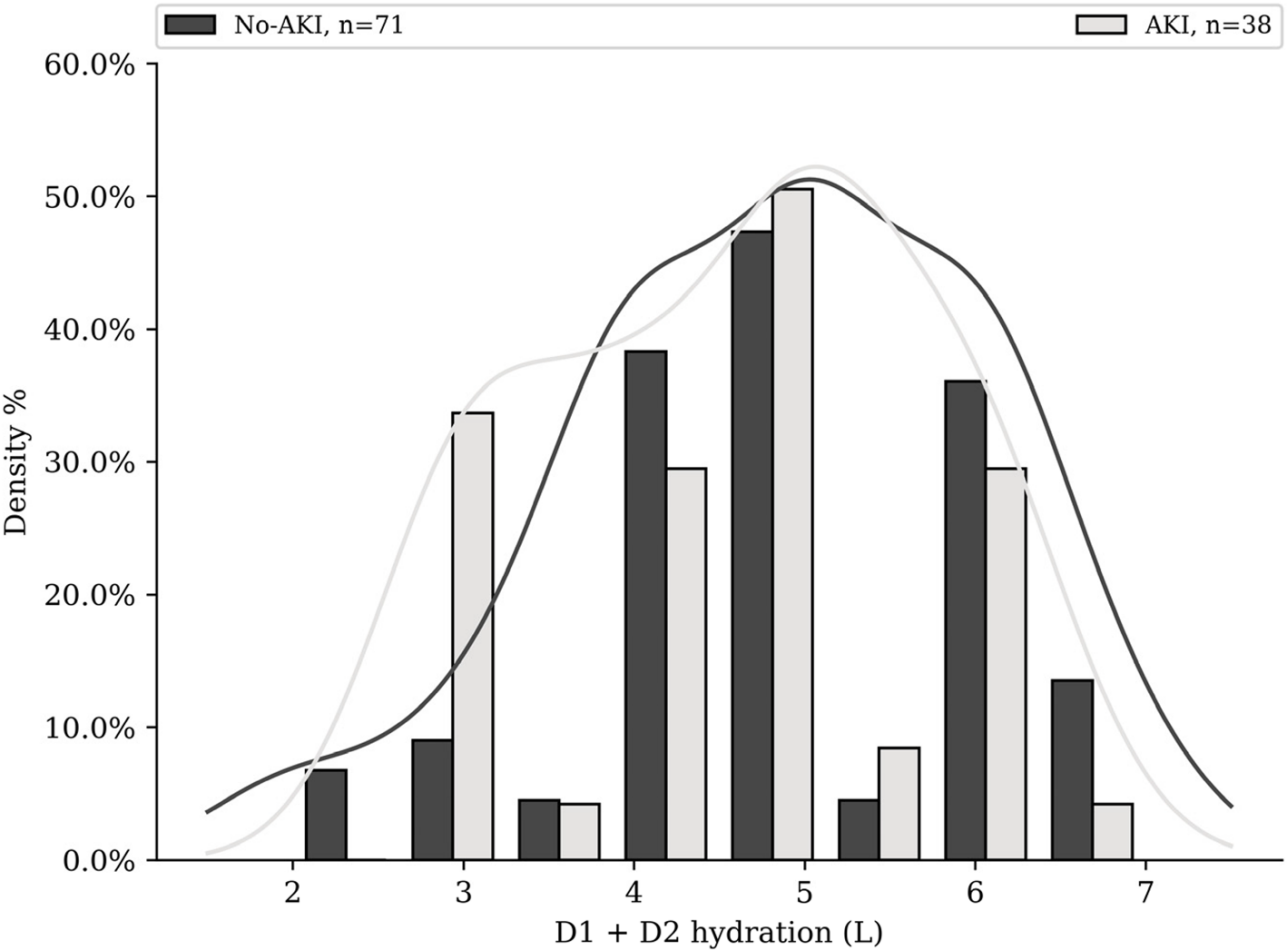
Distribution of hydration volume received by the patients in AKI subgroup and non-AKI subgroup

**Fig. 3:**
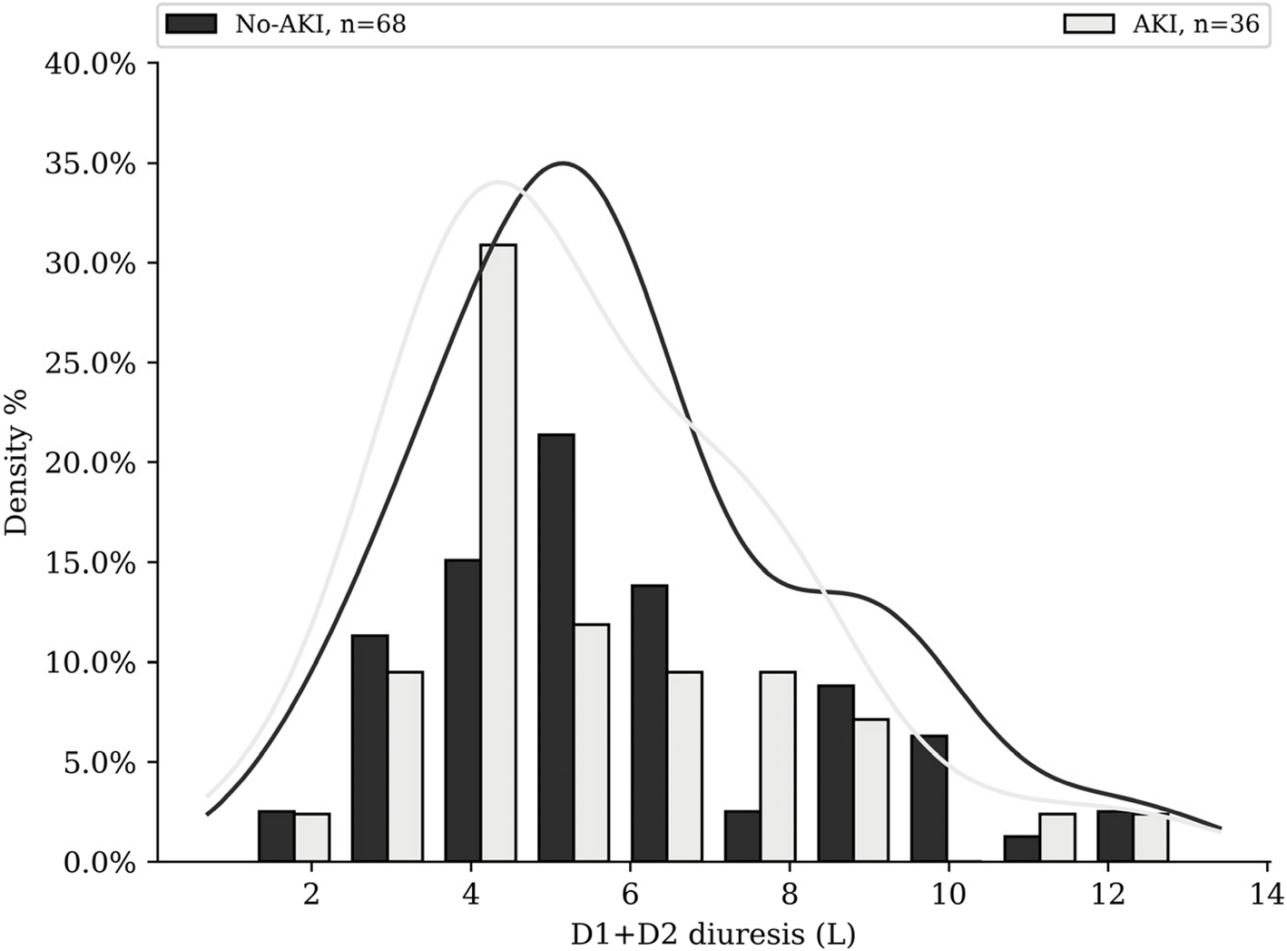
Distribution of urine outputs of each patient’s cycle in AKI subgroup and non-AKI subgroup

**Fig. 4:**
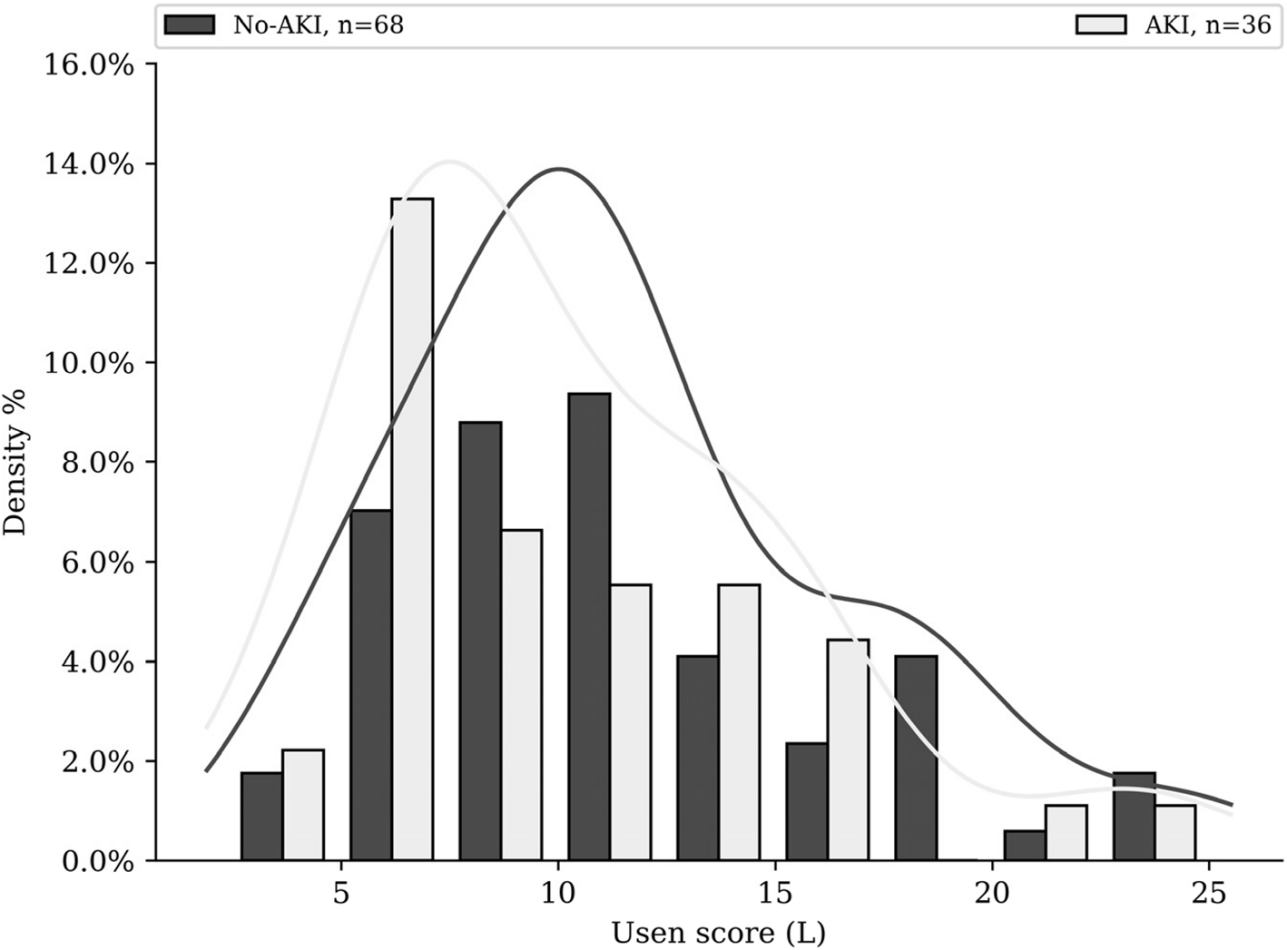
Distribution of ${U}_{sen}$ values of each patient’s cycle according to the AKI variable in AKI subgroup and non-AKI subgroup

Patient’s 110 cycles characteristics and univariate analysis are visible Table 3. Among cycles, 35% lead to at least one ≥ grade 1 AKI. The mean D1 + D2 hydration volume was 4.8 L/48 h (Fig. 2) and mean output was 5.7L/48 h (Fig. 3). ${U}_{sen}$ values distribution is available in Fig. 4.

**Table 3:** Cycles characteristics with and without AKI and analysis

Variable	All (*n* = 110)	AKI cycles (*n* = 38)	No AKI cycles (*n* = 71)	*p*
Wheight loss > 10%, n	51 (46%)	19 (50%)	31 (44%)	0.67
BMI < 18,5, n	21 (19%)	5 (13%)	16 (23%)	0.31
Hypoprotidemia, n	32 (30%)	14 (38%)	17 (25%)	0.25
Dose reduction cisplatin, n	21 (19%)	3 (8%)	18 (25%)	0.039
Dose reduction cetuximab, n	14 (13%)	4 (11%)	10 (14%)	0.77
Cisplatin anteriority, n	16 (15%)	9 (24%)	7 (10%)	0.086
Hypokaliemia, n	11 (10%)	4 (11%)	7 (10%)	1
${U}_{sen}$>8, n	78 (70.9%)	21 (55%)	56 (79%)	0.018
Initital creatininemia, mean (± standard deviation)	62.4 (15.0)	62.5 (13.9)	62.0 (17.3)	0.87
D1 + D2 diurese, mean (± standard deviation)	5.8 (2.4)	5.5 (2.4)	5.9 (2.5)	0.41
Hydration D1 + D2, mean (± standard deviation)	4.8 (1.2)	4.6 (1.1)	4.9 (1.2)	0.28
History of alcohol (> 4U/day) and tobacco abuse (≥ 10 pack-years), n	57 (52%)	23 (61%)	33 (46%)	0.23
cardiovascular impairment, n	52 (47%)	21 (55%)	31 (44%)	0.34
hepatic impairment, n	10 (9%)	4 (11%)	6 (8.5%)	0.74
Metastasis, yes, n	57 (52%)	16 (42%)	40 (56%)	0.22
Enteral nutrition, n	32 (29%)	13 (34%)	19 (27%)	0.55
Concomitant treatments				
Diuretics, n	2 (2%)	1 (2.6%)	1 (1.4%)	1
Cardiac treatments, n	24 (22%)	13 (34%)	11 (16%)	0.03
PPIs, n	74 (67%)	25 (66%)	48 (68%)	1
Metoclopramide, n	30 (27%)	11 (29%)	19 (27%)	0.99
Ondansetron, n	89 (81%)	31 (82%)	58 (82%)	1
Morphine and derivates, n	41 (37%)	17 (45%)	24 (34%)	0.36
Potentially nephrotoxic antibiotics, n	8 (7%)	3 (7.9%)	5 (7%)	1
NSAIDs, n	0 (0%)	0 (%)	0 (%)	1
Number of nephrotoxic or interacting co-medications ≥ 3	40 (37%)	18 (47.4%)	22 (31.0%)	0.14

In univariate analysis we highlighted significant difference between concomitant cardiac treatments (OR = 3.24, [1.26; 8.52], *p* = 0.02), cisplatin dose reduction (OR = 0.166, [0.04; 0.75], *p* = 0.01) and a ${U}_{sen}$ score > 8 (OR = 0.316, [0.133; 0.755], *p* = 0.015) (Tables 3 and 4). No significant difference between age, alcohol and tobacco abuse or previous abused, cardiovascular disease, anteriority of cisplatin treatment, diabetes mellitus, D1 + D2 diuresis > 3.5L (OR = 0.65, [0.272; 1.38], *p* = 0.36) (Table 4) and Body Mass Index (BMI) < 18.5 were found. Concomitant treatments and ≥ 3 nephrotoxic or interacting with cisplatin elimination/excretion co-medication does not show a significant association with AKI (OR = 2.0 [0.89; 4.51], *p* = 0.14).

**Table 4:** Univariate and multivariate logistic regression analysis

Variable	Univariate analysis		Multivariate analysis	
	Odds ratio	*p*-value	Odds ratio	*p*-value
Dose reduction cisplatin	0.166 [0.04; 0.75]	0.01	0.129 [0.0241; 0.687]	0.016
Cisplatin anteriority	2.9 [0.98; 8.61]	0.08	2.55 [0.753; 8.64]	0.13
${U}_{sen}$>8	0.316 [0.133; 0.755]	0.015	0.184 [0.0648; 0.523]	0.0015
Cardiac treatments	3.24 [1.26; 8.52]	0.02	3.18 [1.1; 9.22]	0.033
D1 + D2 diuresis > 3.5 L	0.65 [0.256; 1.32]	0.36		
≥ 3 potentially nephrotoxic concomitant treatments	2.0 [0.89; 4.51]	0.14		

In multivariate analysis, concomitant cardiac treatments (OR = 3.18, [1.1; 9.22], *p* = 0.033) were associated with higher rates of AKI. Reduction of cisplatin dose (OR = 0.13, [0.02; 0.69], *p* = 0.017) and ${U}_{sen}$ score > 8 (OR = 0.18, [0.06; 0.52], *p* = 0.0015) were associated with lower rates of AKI. Anteriority of cisplatin treatment (OR = 2.55, [0.75; 8.64], *p* = 0.13) was not likely to influence AKI risk (Table 4).

## Discussion

In this retrospective study, we highlighted that a ${U}_{sen}$ value > 8/48 h was significantly associated with a lower cisplatin-induced AKI risk in cycles of R/M HNSCCs patients. Higher AKI risk was associated with concomitant cardiac treatments, while reduction of cisplatin dose was negatively associated with AKI risk in multivariate analysis. Patient characteristics such as risk factors, primary tumor location, basal sCr, age, gender and metastatic status were not significant in univariate analysis.

While hydration in the management of cisplatin induced AKI risk has already been extensively evaluated and reviewed by Crona et al. [35], the associated diuresis has been poorly studied. Hyperhydration regimens are basically designed to increase glomerular flow to eliminate cisplatin and avoid its accumulation in the RPCs. Recommendations indicate that the volume of urine should be greater than i.v. hydration [32, 35], given oral intakes. However, several reasons can lead to a mismatch between urine volume and hydration. Initial or current hydration trouble, as chemotherapy nausea and vomiting, was strongly associated with a higher risk of AKI by Vorst et al*.* [23] in a multivariate logistic regression despite adequate hydration in a locally advanced HNSCCs population. Likewise, active alcohol abuse may lead to chronic dehydration, but according to the literature, we did not highlight a significant association [23, 44, 45].

In the ${U}_{sen}$ equation, the discrepancy between urine output and i.v. hydration can refer as the hydration patient status (or “patient hydration responsiveness”). A $\left({HV}_{D1+D2}-{UV}_{D1+D2}\right)<0$ underly a dehydration status or a delayed diuresis. The addition of ${UV}_{D1+D2}$ in the ${U}_{sen}$ calculation represent as faithfully as possible what the kidney is currently filtering and consider all water intakes unlike i.v. hydrate volume. Thus, ${U}_{sen}$ attempted to be an “all in one” score that aims to provide information about hydration status and kidney filtrations capacities of patients by condensing diuresis and i.v. hydration.

The development of this indicator is part of the effort to improve the management of these patients treated with high-dose cisplatin, and highlights the importance of monitoring patients’ initial hydration status more closely. Assessing the volume of oral hydration in the 24 h prior to the cisplatin administration could identify patients at high risk of AKI. However, it would be necessary to admit the patient to hospital the day before the injection in order to quantify these volumes precisely. Thus, nursing staff could encourage patients to drink or, if they are unable to swallow, to initiate i.v hydration. This would involve increased costs [46]; which current healthcare systems are unable to accommodate. Some centers offer an alternative consisting of pre-hydrating patients at home using home elastomeric infusion pumps. This ensures optimal hydration regardless of the patient’s history and risk factors, but requires nursing care for the implementation. Further studies could be carried out to discern a suitable and efficient intakes measurement method.

In practice, patients with a ${U}_{sen}$ score < 8/48 h could benefit from intensified clinical and biological monitoring as well as measures to increase glomerular filtration as additional hydration combined with forced diuresis (with mannitol or furosemide) to enhance urine flow to limit cisplatin accumulation in the RPCs. The use of diuretics or mannitol are controversial methods that seems to be relevant for high-dose cisplatin but carry a major risk of dehydration, especially if losses are not compensated [35]. However, considering the reduce in urinary cisplatin concentration demonstrated in vivo a dose-dependent decrease in the risk of nephrotoxicity by forced diuresis [47, 48], this method combined with extra hydrates should be considered in patients with delayed or insufficient diuresis. Thus, a cisplatin-related AKI predictive score could be strategic in order to trigger the forced diuresis regimen, considering that cisplatin-related AKI occurs several days after administration [35]. Moreover, the antiemetic protocol must also be carefully considered in patients with swallowing disorders. The use of liquid pediatric forms should be considered, if not to increase other treatments that may interact with cisplatin elimination.

According to the literature, the development of a new tool in the management of AKI in these cisplatin-treated patients is even more important as the incidence of AKI is markedly increased in patients presenting head and neck cancer [23, 42, 45]. While some risk factors seem to stand out, it is still unclear why these cisplatin treated patients have such a high AKI incidence. Considering the tumor location and the radiotherapy toxicity, HNSCCs patients suffer from an increased risk of malnutrition (weight loss and hypoalbuminemia) [49, 50]. Weight loss or BMI remain unclear about the association with cisplatin-AKI risk [41, 44, 45]. To the contrary, as cisplatin binds irreversibly to blood albumin [51], albumin blood level reduction showed a positive association with AKI risk in multivariate analysis [44, 52] so was included in the risk prediction model of AKI developed by Motwani et al. [28]. However, in smaller HNSCCs cohort studies, albumin does not appear to be associated with AKI risk, although albumin cut-off values used, up to 4 g/dl, lack clinical meaning [42, 53]. We could not evaluate this variable due to insufficient data.

In this study, we highlighted a significant association with concomitant cardiac treatments and AKI risk in univariate and multivariate analysis of the cycles. Converting enzyme inhibitor, angiotensin II receptor blockers and calcium channel blockers have previously demonstrated an increase of cisplatin-related AKI [53–55]. Meanwhile, we showed a positive trend with cardiovascular disease history without reaching significance as reported in several studies [41, 45, 53]. Other articles consider hypertension or cardiovascular disease associated with cisplatin AKI risk factor [23, 28, 55, 56], but cardiac concomitant treatment administration was uncommonly evaluated. These cardiac treatments are known to affect afferent or efferent renal arterioles tonus and many interfere with OCT2 and MATE [57] which may alter with cisplatin elimination. In this sense, Takeuchi et al. [54] reported a cumulative cisplatin related AKI-risk with the concomitant administration of several classes of antihypertensive treatments. However, the administration of antihypertensive therapies implies patient is suffering from an underlying hypertensive or cardiovascular disease, and the addition of extra cardiac treatment suggest a more serious trouble. Nevertheless, a combining risk is conceivable and further clinical trials should be performed to identify whether a predominant factor exists.

Certain MATE1/2 K and OCT2 interacting treatments may have a significant impact on the elimination of cisplatin if glomerular filtration is insufficient. OCT2 inhibitors such as proton pump inhibitor (PPI) or ondansetron, a MATE1/2 inhibitor [57] did not show any association with AKI risk. As well, concomitant ≥ 3 nephrotoxic or modulating with cisplatin elimination comedication provided identical results. This may be explained by the method used to collect the intakes, which only includes the first 2 days of the cycle and does not assess the patient’s long-term intakes or pre-cycle exposure.

## Limitations

Our study presents many limitations considering the retrospective design. The veracity and accuracy of diuresis data could not be verified and although these data were available in the records, some appeared inconsistent. To limit these inconsistencies, we chose to average the diuresis over only the first 48 h post cycle. Given the extended inclusion time frame, we cannot guarantee consistent patient management over this period. The analysis by cycle received may introduce a bias, given that some patients developed several AKIs during their stay, even though patient characteristics are similar and biological and clinical factors do not differ before each cycle. A certain proportion of patients with cardiac disorders did not receive cardiac treatment during their cure, so it is likely that they had interrupted their intake during this period.

Two patients included in our study presented a Creatinine Clearance (calculated with Cockcroft-Gault equation) < 60 ml/min prior to their first and only cycle. Considering the cisplatin Summary of Product Characteristics, they should not have received the cisplatin based EXTREME.

Finally, this new score evaluation should be duplicated in a prospective larger cohort considering the limited number of patients included.

## Conclusion

The prevention of high dose cisplatin-induced AKI is a major issue in the management of head and neck cancer presenting patients. Through the evaluation of a new monitoring indicator called ${U}_{sen}$ combining diuresis and hyperhydration, we were able to identify a cut-off value associated with the occurrence of cisplatin-related AKI. ${U}_{sen}$ should be considered in further clinical trials to assess its relevance in the prevention of AKI by providing an indicator to trigger a specific salvage protocol.

## Data Availability

The datasets used and/or analyzed during the current study are available from the corresponding author on reasonable request.
